# Perspectives on the Performance of the Ethiopian Vaccine Supply Chain and Logistics System after the Last Mile Delivery Initiative: A Phenomenological Study

**DOI:** 10.4269/ajtmh.23-0622

**Published:** 2024-04-02

**Authors:** Samson Gebremedhin, Fisseha Shiferie, Dawit A. Tsegaye, Wondwossen A. Alemayehu, Tamiru Wondie, Solomon Zeleke, Belete Alebachew, Jen Donofrio, Frank DelPizzo, Kidist Belete, Gashaw Andarge Biks

**Affiliations:** ^1^ School of Public Health, Addis Ababa University, Addis Ababa, Ethiopia;; ^2^ Project HOPE, Ethiopia Country Office, Addis Ababa, Ethiopia;; ^3^ Project HOPE Headquarters, Washington, District of Columbia;; ^4^ Ministry of Health, Addis Ababa, Ethiopia;; ^5^ Bill and Melinda Gates Foundation, Seattle, Washington;; ^6^ U.S. Agency for International Development, Ethiopia, Addis Ababa, Ethiopia

## Abstract

Uninterrupted availability of vaccines requires a robust vaccine supply chain and logistics system (VSCLS). With special focus on remote and underserved settings, we assessed the reach and bottlenecks of the Ethiopian VSCLS after the initiation of the last mile transition. We explored the perspectives of key stakeholders using a qualitative phenomenological study. More than 300 in-depth interviews and 22 focus group discussions were conducted. The study was sequentially implemented over two phases to understand the bottlenecks at national and regional (Phase I) and lower (Phase II) levels. After the transition, the Ethiopian Pharmaceutical Supply Service started supplying vaccines directly to health facilities, bypassing intermediaries. The transition reduced supply hiccups and enabled the health sector to focus on its core activities. However, in remote areas, achievements were modest, and health facilities have been receiving supplies indirectly through district health offices. By design, health posts collect vaccines from health centers, causing demotivation of health extension workers and frequent closure of health posts. Challenges of the VSCLS include artificial shortage due to ill forecasting and failure to request needs on time, lack of functional refrigerators secondary to scarcity of skilled technicians and spare parts, and absence of dependable backup power at health centers. Vaccine wastages owing to poor forecasts, negligence, and cold chain problems are common. The VSCLS has not yet sustainably embraced digital logistics solutions. The system is overstrained by frequent outbreak responses and introduction of new vaccines. We concluded that the transition has improved the VSCLS, but the reach remains suboptimal in remote areas.

## INTRODUCTION

Vaccination is one of the most cost-effective public health interventions. Annually, vaccines save nearly 4.5 million lives with an economic return of $26 for a dollar invested. Yet globally, 18.2 million children are zero-dose, and 25 million are undervaccinated.^1^ Just 10 countries, including Ethiopia, contribute to more than 60% of the global zero-dose burden.^2^ In Ethiopia, 30% of infants, equivalent to more than a million children, are unvaccinated.^2^

An uninterrupted supply of vaccines is essential for a successful immunization program.^3^ Yet, in many countries a vaccine supply chain and logistics system (VSCLS) has received little attention. The underperformance of the logistics system is a major constraint to achieving high coverage in many low- and middle-income countries (LMICs).^4^^,^^5^ Vaccine supply chain and logistics systems represent the cycle of vaccine acquisition, storage, distribution from national hubs to service delivery points, waste management, and timely requests for supply.^3^^,^^6^ An effective VSCLS requires a robust system, functional personnel, and appropriate technologies.^6^ The Immunization Agenda 2030 and the Global Vaccine Alliance (GAVI) 5.0 “leave no one behind with immunization” strategies have recently identified effective VSCLSs as a global priority.^7^

In LMICs, VSCLSs are under increased pressure to accommodate the introduction of new vaccines and rapidly growing needs.^3^^,^^8^ Recently, several countries, including Ethiopia, added new vaccines to their Expanded Program on Immunization (EPI) schedules.^9^ The COVID-19 pandemic also affected VSCLSs globally.^10^ Conversely, digital technologies, cold chain innovations, and temperature monitoring systems are becoming available to meet ever-increasing complexities.^11^ The VSCLS in many LMICs may benefit from systematic redesigning.^5^

The Ethiopian VSCLS is directed by the Ethiopian Pharmaceutical Supply Service (EPSS), an independent agency affiliated with the Ministry of Health (MoH). The EPSS, via its 19 regional hubs, supplies more than 4,000 hospitals and health centers. Recently, multiple vaccine supply chain transformations have been initiated in Ethiopia.^12^ Previously, EPSS distributed vaccines indirectly through regional health bureaus (RHBs) and other intermediaries in the health system. However, in the mid-2010s, the “last mile vaccine delivery” approach by which EPSS directly supplies health facilities was initiated, and in the late-2010s, it was scaled up.^12^ The MoH envisages increasing the proportion of health centers and hospitals that directly receive vaccines from EPSS hubs to 90% by 2025.^13^

The vaccine supply in many countries may benefit from supply system redesigning, including bypassing intermediaries.^5^ Analysis of the vaccine logistic systems of 57 LMICs indicated that the majority of the systems were composed of four levels of intermediaries, indicating the need for simpler and shorter supply chain systems.^14^ A randomized controlled trial for improving the supply chain for essential drugs in Zambia indicated that a more direct distribution system, compared with the traditional three-level system, reduced the frequency of stockouts.^15^ Studies in Niger and Nigeria also reached similar conclusions.^16^^,^^17^

The effectiveness of the VSCLS of Ethiopia, a country that has one of the highest zero-dose burdens in the world, has not been systematically investigated. Although multiple studies in other LMICs have indicated the benefits of supply system redesigning, including bypassing intermediaries, the impact of the last mile delivery system in Ethiopia has not been documented. The few available studies have not looked into the holistic system^18^ or were limited to health workers’ behavior.^19^ The national Service Availability and Readiness Assessment (SARA) survey conducted in 2018 indicated that only 28–30% of health facilities in the country had the essential vaccines, and only 30% of them owned refrigerators.^20^

This work aimed to assess the reach of and bottlenecks to end-to-end delivery of the Ethiopian VSCLS after the initiation of the last mile transition, with a special focus on remote and underserved settings in Ethiopia.

## MATERIALS AND METHODS

### Overview.

This qualitative study was nested within a large study that Project HOPE, The People-To-People Health Foundation Inc., undertook in 2022. The prime study intended to understand the barriers to reaching zero-dose children in underserved settings in Ethiopia, and the findings have been presented elsewhere.^21^ Project HOPE is an international U.S.-based nongovernmental organization (NGO) that implements humanitarian and health development programs in more than 25 countries, including Ethiopia.

### Study design.

We conducted a phenomenological qualitative study to understand the bottlenecks of the VSCLS. We explored the perspectives of key players in the system, including healthcare decision-makers at all levels, EPSS officers at central and regional hubs, representatives of relevant NGOs, frontline health workers, and EPI focal persons deployed at primary healthcare units (PHCUs). We also explored the viewpoints of influential community members, voluntary community workers, and caregivers.

We preferred a qualitative, rather than a quantitative, study because a qualitative phenomenological study is more robust for understanding peoples’ experience with a phenomenon (in this case, various stakeholders of the vaccine logistic system) and identifying barriers and enablers to implementation of the program.

Data were collected primarily through key informant interviews (KIIs). Focus group discussions (FGDs) were also conducted with caregivers. The study was sequentially implemented over two phases to explore bottlenecks at national and regional (Phase I) and lower (Phase II) levels (Table 1).

**Table 1:** Description of the two phases of the study

Phase	First Phase	Second Phase
Purpose	To Capture the Perspective of Top-Level Policy and Decision-Makers of the Health System and Core Partners Engaged at a Higher Level of the System To Identify Underserved Settings and Population for the Second-Phase Study	To Gain a Deeper Understanding into the Perspective of Middle- and Lower-Level Managers, Health Workers, and Partner NGOs Working at a Lower Level To Explore the Viewpoints of Community Members, Including Caregivers
Time	February–March 2022	May–June 2022
Participants	Key informants from the MoH, RHBs, and EPSS hubs Immunization Focal Persons from Core Partner Organizations	Key informants from Zonal and Woreda Health Offices Health Workers from Primary Health Care Units Caregivers and Community Members
Settings	MoH, all RHBs, and EPSS Hubs	Selected Underserved Zones and Districts in All Regions of Ethiopia
Data Collection Approach	KII Interviews	KIIs and FGDs
Number of Interviews	98 In-Depth Interviews	229 KIIs and 22 FGDs

EPSS = Ethiopian Pharmaceutical Supply Service; FGD = focus group discussion; KII = key informant interview; MoH = Ministry of Health; NGO = nongovernmental organization; RHB = regional health bureau. Data were collected in February to March (Phase I) and May to June (Phase II) 2022. The period coincides with the end of the COVID-19 pandemic and outbreak of widespread political instability in Ethiopia.

### Study context.

The Ethiopian healthcare system is organized into three tiers: primary, secondary, and tertiary. A PHCU constitutes a primary hospital (1 per 60,000–100,000 population), health centers (1 per 15,000–25,000 population), and health posts (1 per 3,000–5,000 population).^22^ In 2020, Ethiopia had 17,550 functional health posts, 3,735 health centers, and 353 hospitals.^23^ At the time of the study, Ethiopia was subdivided into 11 federal states and two chartered cities, each with a decentralized health system having an RHB, zonal health departments (ZHDs), and woreda/district health offices (WoHOs).^11^

**Figure 1.**
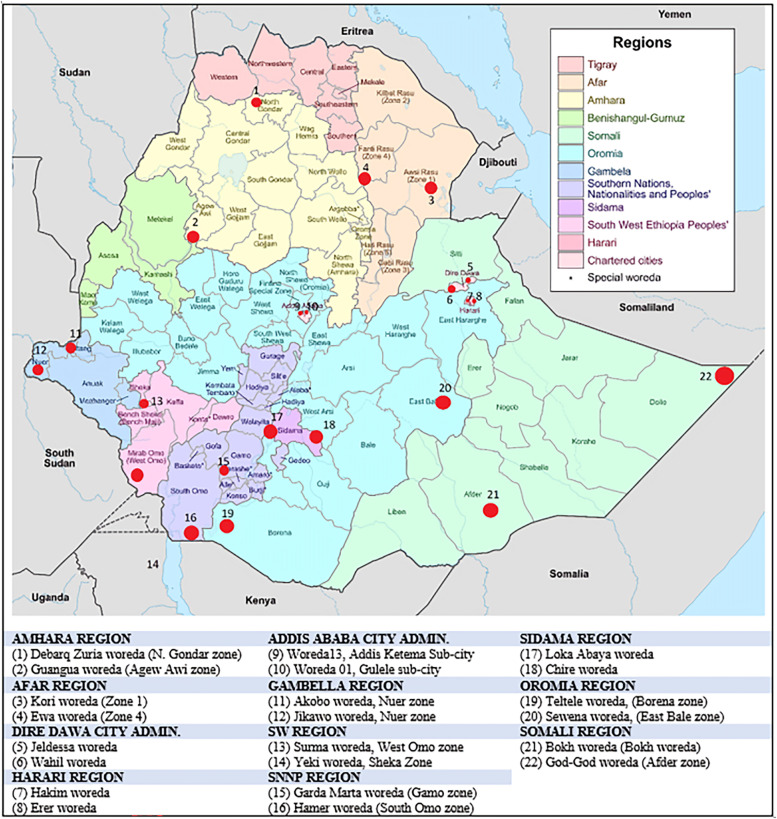
Localities where the second-phase study was conducted.

During the first phase of the study, we explored the vaccine logistics system in all regions except Tigray, where active conflict was ongoing. In the second phase, we explored how the system performs at zonal and woreda levels, with a special focus on underserved settings including hard-to-reach and conflict-affected districts. In the second phase study, we included districts from all regions except Tigray and Benishangul Gumuz (Figure 1).

We focused primarily on vaccines that are routinely provided to infants. The Ethiopian EPI schedule recommends the Bacille Calmette-Guerin (BCG) vaccine and oral polio vaccine (OPV) -0 at birth, the Pentavalent, pneumonia conjugate vaccine (PCV), and subsequent doses of OPV at 6, 10, and 14 weeks, the rotavirus vaccine at 6 and 10 weeks, the inactivated poliovirus vaccine at 14 weeks, and the measles vaccine at 9 and 15 months.

### Sampling strategy.

Key informants who have firsthand knowledge of the Ethiopia VSCLS were purposively selected. In the first phase, 98 informants at national and regional levels, including respondents from the MoH, RHBs, central and regional EPSS hubs, and Ethiopian Public Health Institute and EPI program leads at multilateral agencies and NGOs, were interviewed.

The second phase of the study was conducted in 22 remote and underserved districts purposively drawn from 11 regions and chartered cities of Ethiopia for having low vaccination coverage. Districts from Tigray and Benishangul Gumuz regions are not represented owing to security problems at the time of the study.

In this phase, we completed 229 KIIs and 22 FGDs. Key informants included managers and EPI coordinators at ZHDs, WoHOs and relevant NGOs and frontline health workers at PHCUs, including health extension workers (HEWs). We also interviewed influential community members and volunteer community health workers and organized FGDs with caregivers of children 12–23 months of age. In the FGDs, heterogeneous sampling that included caregivers with optimal and suboptimal vaccination utilization was applied. The list of key informants is provided in Supplemental Table 1.

### Data collection procedures.

The KIIs and FGDs were facilitated using semi-structured guides based on 8–10 open-ended questions and subsequent probing prompts. Distinct interview guides were developed for different interviewees. For instance, the guide for EPSS officers was limited to the VSCLSs from the central EPSS hub to health facility levels. The guides for health officials and workers focused on the situation within the healthcare system. The interview guides used for community-level respondents explored implications of the logistics system on the service received at the ground level. Ahead of the field engagement, all the guides were piloted and later validated in a stakeholders meeting. The “reach” of the VSCLS before and after the initiation of last mile delivery was assessed by asking the respondents to compare and describe their experiences with vaccine stockout and supply interruption before and after the transition.

Interviews were conducted face-to-face by trained interviewers and notetakers at the workplace of the informants in the absence of observers. The study design evolved over the phases, and the findings of the first phase were used to fine-tune the subsequent phase. Data were gathered until information saturation was achieved, as perceived by the researchers. However, as indicated in Supplemental Table 1, a minimum of 10 interviews per sampling “grid” were conducted. All interviews were audio recorded and later transcribed and translated into English verbatim. Field notes were completed to provide contextual information.

#### Ensuring trustworthiness.

We applied the four-dimensions criteria (credibility, transferability, dependability, and confirmability) considered to be markers for the trustworthiness of qualitative research.^24^ To ensure credibility, we represented all regional states and city administrations of Ethiopia. Furthermore, we assessed the perspectives of the entire structure of the health system and EPSS by including key informants from every level of the system. We maximized our field engagement by implementing the study over two phases. We provided extra attention to remote and underserved settings, where supply bottlenecks were likely to be evident. Investigator triangulation was achieved by engaging two analysts in data interpretation. We triangulated and complemented the perspectives of diverse informants, including decision-makers in the health system, EPSS officers, partner organizations, health workers, and community members.

Two-staged checking processes, first with research team members and then with those who provided the data, were used to assess the credibility, transferability, and confirmability of the study. To this end, two validation workshops with a sample of key informants from the MoH, RHBs, partner organizations, and the EPSS were organized. Peer debriefing was also done to improve the dependability of the study. Discrepancies were solved by going through the transcripts and coding again. Transferability was promoted by presenting the key findings along with pertinent contextual data, including study regions and settings.

#### Reflexivity.

We continually incorporated reflexivity in the research process, from interview–guide development to coding and data interpretation. The researchers were all outsiders to the VSCLS, so they had little or no preformed biases. However, because the study focused primarily on remote and underserved settings in Ethiopia, we had an underlying assumption that the VSCLS might not robustly function there. However, we tried our best to do “bracketing” and set aside this assumption. We engaged with officials from the MoH throughout the research process; however, that did not affect our data interpretation. The researchers were not involved in actual data collection but provided regular feedback to the interviewers during their field engagement.

### Data analysis and interpretation.

#### Data management.

A thematic approach was used to analyze the data. Two investigators familiarized themselves with the data, independently carried out manual data-driven inductive coding, and generated tentative themes. Later, they jointly finalized the themes. Data coming from the two phases were separately analyzed; however, we reexamined earlier insights against emerging insights in the subsequent phase. The report was synthesized in a logical sequence according to the themes. Pertinent quotes are provided to underpin data interpretation and demonstrate that the conclusions have emanated from the data. Data coming from FGDs and in-depth interviews are presented in an integrated manner depending on the emerging themes. Table 2 summarizes the analytical themes and sub-themes.

**Table 2:** Codes, subthemes, and themes

Codes	Subthemes	Themes
Improvements after the Transition	Success Stories	The Last Mile Delivery Transition
Implications of the Transition	Challenges	
Incomplete Transition		
Procurement Delays	Stockout at National Level	Supply Chain Hiccups
National Stockout		
Conflict	Supply Interruptions	
Remoteness		
Climatic Factors		
COVID-19 Outbreak	Overstretched Supply System	
Introduction of New Vaccines		
Vaccination Campaigns		
Digital Technologies	Opportunities for the VSCLS	
Negligence	Failure to Request Vaccine Needs on Time	Timely Request for Adequate Vaccines
Artificial Shortage		
Ineffective Trainings		
Forecasting Problems	Incorrect Estimation of Needs	
Faulty Population Denominators		
Unpredictable Target Population change		
Expansion of Cold Storage Capacity	Success Stories	Challenges and Success of the Cold Chain System
Mass Distribution of Refrigerators		
Refrigerator Maintenance Campaigns		
Deployment of Biomedical Technicians		
Training of Health Workers		
Availability of Fridge-Tag System		
Cold Storage at Hubs	Cold Storage and Transportation of Vaccines	
Cold Storage at Districts and Health Facilities		
Shortage of Refrigerated Trucks		
Shortage of Refrigerators at Health Posts	Shortage of Functional Refrigerators at Health Facilities	
Shortage of Spare Parts		
Shortage of Skilled Technicians		
Maintenance Problems		
Not Following OVW Policy	Open Vial Wastage	Vaccine Wastage
Negligence of Health Workers		
Excess Supply During Campaigns		
Challenges with the Fridge-Tag System	Closed Vial Wastage	
Distribution of Near-Expire Vaccines		
Cross-Hub Transfer	Efforts to Reduce Vaccine Wastage	
Training on EVM		
Temperature Monitoring System		

EVM = effective vaccine management; OVW = open vial wastage; VSCLS = vaccine supply chain and logistics system.

## RESULTS

### Last mile vaccine delivery: Success stories and challenges.

Prior to the last mile delivery initiative, RHBs used to distribute vaccines within their boundaries. At that moment, supply hiccups were very common, as the RHBs were inefficient at distributing vaccines. After the transition, the EPSS commenced supplying vaccines from its regional hubs directly to hospitals and health centers, bypassing multiple hierarchies of the health system including RHBs. According to key health workers from health centers and health posts, over the transition period the uninterrupted availability of different antigens improved. According to health officials, the transition advanced the national VSCLS and enabled the health sector to focus on its core activities. Different success stories of the initiative, including improvement in effective vaccine management (EVM), were reported.

“Earlier, the effective vaccine management score in the region [Benishangul Gumuz] was very low. Now, it has considerably improved” (key informant from Assosa EPSS hub).

“After the transition there is no problem of distributing the vaccines to the districts” (key informant from Oromia RHB).

From the EPSS’s perspective, the initiative has increased its workload; however, the agency sustained the burden by expanding and capacitating regional hubs, designating regional EPI focal persons, and introducing standard operating procedures. In general, regional EPSS hubs are discharging their duty optimally and have established a good working relationship with the health system. As recognized by EPI officers from the health system and the EPSS, the VSCLS has received greater attention than other medical commodities within the EPSS.

“The existing logistic system for vaccines is even better [than] the system for other medical supplies. Within EPSS, special attention has been given to vaccines including assignment of vaccination focal persons” (key informant from the EPSS).

Nonetheless, the transition is not complete in remote areas, where most health centers are still receiving their supplies indirectly through WoHOs. Furthermore, the initiative has not reached health posts; as a result, HEWs continue to collect their supplies from health centers. This has been cited as a demotivating task by many HEWs. From the caregiver’s perspective, waiting for the HEW to collect the vaccines from the health center was reported as a cause of service dissatisfaction. Especially in developing regions (Somali, Afar, Benishangul Gumuz, and Gambella) and other remote settings where the health system is fragile, the weak health center–health post linkage has compromised the availability of vaccines at the grassroots level. Health centers also lack the resources to distribute medical supplies to their satellite health posts.

“Now [after the transition], there is no problem of distributing the vaccines to the districts. Now the problem is dispensing it from the districts to health facilities” (key informant from Oromia RHB).

“But we don’t have any means of transporting vaccines from health centers to health posts” (key informant from Teltele WoHO, Oromia region).

The last mile delivery has not been free from hitches. Major challenges from the EPSS’s perspective include a shortage of refrigerated trucks and unmanageably large catchment areas for some hubs (e.g., Somali region). In developing regions and remote districts, owing to the bad road conditions, available trucks frequently break and are out of service for months. Expanded Program on Immunization focal persons at health centers also criticized the EPSS for distributing vaccines using drivers who lack technical expertise on how to handle vaccines.

“Usually, drivers who don’t have any technical knowledge bring the vaccines. If you tell them the supply is inadequate or the vaccine vial monitor (VVM) is not OK, they don’t understand anything” (key informant from a primary hospital, Amhara region).

### Situation of the vaccine supply chain.

#### Procurement and delivery at the national level.

According to key informants from the MoH, Ethiopia relies heavily on the GAVI for its vaccine needs. UNICEF purchases and delivers the vaccine on behalf of the government. However, in response to GAVI’s funding requirements, Ethiopia recently initiated a co-financing scheme for a few antigens. According to officials from the MoH, the co-financing has demonstrated the commitment of the government for EPI. Conversely, informants from multilateral agencies indicated that vaccines are still considered donor-supplied commodities, and the government’s sense of ownership is modest. Many informants concurred that donor dependency is a major threat to the sustainability of EPI in Ethiopia, including the VSCLS.

“It has been more than 40 years since Ethiopia started the EPI. But the program is still donor dependent and does not stand by itself” (key informant from an RHB).

#### Stockout and supply interruptions.

Respondents from the RHBs and WoHOs reported that vaccine supply interruptions have been encountered occasionally as a result of national stockouts or procurement delays. Nationwide stockouts have recently been reported for BCG and Rota vaccines. In the districts of Amhara, Afar, Southern Nations, Nationalities and Peoples (SNNP), and Sidama regions that we studied, the EPSS recently supplied an inadequate number of vaccines owing to national stockouts. As reported by caregivers and influential community members, children sometimes missed their vaccines because of transient shortages.

“Sometimes children go to the health post. But health workers send them back by saying, ‘vaccines are over’” (an influential community member, Teltele woreda, Oromia region).

“If we get BCG, we may not have Penta. When we get Penta, then…” (key informant from a health center in Surma woreda, Southwest [SW] region).

#### Vaccine supply in remote areas.

Although vaccine supply hiccups are encountered less frequently at the national level, the situations in developing regions and remote districts are less promising. In such settings, supply interruptions for a month or two are common. In distant areas of the SNNP and SW regions, the EPSS frequently has failed to reach remote districts and has “dumped” its supplies at zonal centers or towns in neighboring districts. For many WoHOs, collecting vaccine supplies from elsewhere was not been feasible owing to a shortage of vehicles or budget.

“EPSS dumped our supply at Kemba district. To collect it from there, we need vehicle, fuel and per diem for drivers” (key informant from Gardamarta WoHO, SNNP region).

Poor roads and adverse weather conditions such as flooding hamper the uninterrupted availability of vaccines in the remote districts of the Gambella and SW regions. So far, the EPSS has not established alternative mechanisms to road transportation for reaching remote areas. Accordingly, the system lacks the agility to adapt to natural or manmade disasters.

“Especially during the rainy season, the entire districts of the zone except one will be [cut off] due to the flooding” (key informant from Neur ZHD, Gambella region).

#### Vaccine supply in conflict-affected areas.

Recently, interethnic conflicts have become common in Ethiopia and have caused frequent medical supply interruptions. During the study, an active political conflict was ongoing in northern Ethiopia and seriously limited the availability of vaccines in the Tigray, Amhara, and Afar regions. The conflicts in western and southern Oromia and other pocket areas of the SNNP and SW regions had the same impact. The EPSS attempted to reach some of the conflict-affected areas through chartered flights, but that was not affordable for the agency.

“For the last 3–4 months we have not received any vaccine because of the road closure due to the conflict. We have been using the supplies that we have at hand. Now, almost four or five antigens are over” (key informant from Assosa EPSS hub).

#### Overstretched vaccine supply system.

According to key informants from the EPSS, the MoH, and RHBs, mass vaccination campaigns against the recent COVID-19 pandemic overstretched the national vaccine logistics system and compromised the distribution of routine vaccines. But in the post-COVID era, the situation has improved. Introduction of new vaccines into the immunization schedule and frequent outbreak-response campaigns have also affected the system. The recent cholera and monovalent oral poliovirus vaccination campaigns were cited as challenging experiences by EPSS officers.

#### Logistics of other vaccine-related supplies.

Previously, imbalances in the supply of vaccines and other related supplies happened frequently; however, nowadays such problems have become rare owing to bundled supplies of vaccines and related commodities. In most settings that we studied, major shortages of syringes, diluents, vaccine carriers, and safety boxes have not been encountered. However, sporadic scarcity of vaccination cards, safety boxes, vaccine boxes, and ice bags has been reported.

### Demand forecasting and timely request for vaccines.

#### Forecasting vaccine needs.

Districts and health facilities forecast their vaccine needs based on estimated catchment population size and population conversion factors. According to EPI coordinators at RHBs, WoHOs, and health centers, faulty conversion factors and outdated census statistics frequently cause over- or under-forecasting. This has been identified as a major challenge for vaccination planning in all settings, but it is more evident in developing regions and pastoralist communities. At the community level, HEWs are expected to conduct a head count of eligible children within their catchment area; however, according to respondents from the communities, this has not been done regularly because of demotivation, workload, and lack of accountability. In major cities such as Addis Ababa, additional reasons, including massive rural-urban migration and internal displacements secondary to urban restructuring, have made vaccine forecasting problematic.

#### Timely request for vaccines.

According to key informants from the EPSS, the failure of WoHOs and health facilities to submit vaccine requests on time is a major cause of artificial shortages. This has been reported across the regions, but because of inaccessibility, it occurs more commonly in developing regions and remote districts. Late or incomplete reporting, apparently due to negligence, was also frequently mentioned. Consequently, health facilities miss the supply for at least a month because the EPSS will not provide vaccines without formal requests. To mitigate the problem, EPSS hubs and RHBs have organized trainings for health workers, but that has not been very effective owing to staff turnover and demotivation.

“We provided trainings on timely reporting of vaccine needs. But, due to staff turnover, the outcome has not been satisfactory” (key informant from Hawassa EPSS hub).

#### Digital solutions to improve vaccine logistics information system.

With the support of a partner organization, a mobile phone–based stock management tool called mBrana was introduced by the EPSS. The initiative was implemented at scale, and the system facilitated the vaccine logistics system at all levels, including WoHOs and health facilities. As reported by EPI focal persons at the EPSS and WoHOs, the application was well accepted by the end users, simplified the vaccine information system, and improved the availability of vaccines at the health center level. Unfortunately, the initiative has not been sustained beyond the end of the project because of lack of a robust exit strategy, including a shortage of information technology supplies and technicians at the lower level of the system.

“Recently an NGO initiated a mobile phone–based application that simplified the vaccine logistics system at all levels. The initiative went well for a few years, but later phased out” (vaccination focal person, EPSS regional hub).

### The cold chain system.

#### Success stories.

Over the recent years, the cold storage capacity at central and regional EPSS hubs has expanded considerably. The availability of refrigerators at health posts has also improved owing to mass distribution of solar direct drive (SDD) refrigerators. The increasing availability of biomedical technicians and the implementation of frequent refrigerator maintenance and inventory campaigns are seen as positive moves. Reportedly, a large number of health professionals received training on EVM and preventive maintenance of refrigerators. The topics have also been introduced into the Integrated Refresher Training curriculum designed for HEWs.

#### Cold storage capacity.

According to key informants from the EPSS, central and regional hubs have large cold storage capacities that can accommodate the expanding needs of the country. However, WoHOs and health facilities, particularly in developing regions, are confronted with limited storage capacity. Regional EPSS hubs have the option of sending extra supplies to health facilities for various reasons (e.g., anticipating supply interruption), but they cannot do so because of limited storage capacity at the health facilities. At health posts, the capacity of SDD refrigerators is also limited. In health centers, it is common for other medical commodities such as oxytocin to be stored with vaccines to further limit storage capacity.

“We need at least four refrigerators, but we only have two. We do not have adequate storage capacity to receive all the vaccines that we need” (key informant from Chire WoHO, Sidama region).

#### Shortage of refrigerators at health facilities.

The availability of functional refrigerators at health posts remains unsatisfactory nationwide. The situation in developing and newly established regions is more pressing. In the conflict-affected areas of the Amhara and Afar regions, mass vandalization of refrigerators and other medical equipment has recently occurred. In health posts that do not have refrigerators, routine static vaccination is not feasible, and the service is implemented primarily through irregular outreach activities.

“Out of the 33 health posts in our district only seven have solar refrigerators” (key informant from Debariq Zuria WoHO, Amhara region).

“Regular vaccination service is not provided in many of our health posts because they do not have refrigerators” (key informant from Gardamarta WoHO, SNNP region).

#### Maintenance of refrigerators: Shortage of spare parts and skilled biomedical technicians.

Generally, refrigerators frequently stay out of service because of problems that can easily be prevented (e.g., burnout secondary to a power surge). In settings where refrigerators are available, they are not handled with care, as health workers are not well sensitized on the preventive maintenance procedures needed. In general, broken fridges are not being maintained in a timely fashion because of an inadequate supply of spare parts, a shortage of skilled biomedical technicians at the district level, and bureaucratic administrative procedures. As reported from the Somali and Sidama regions, some health posts have already stopped providing vaccination service owing to broken refrigerators.

“Most health posts or health centers have non-functional refrigerators. Especially when the SDD refrigerators fail, they remain out of service for a long time due to lack of spare parts and qualified technicians” (key informant from Gambella RHB).

*“Once a refrigerator is broken, it is not likely that it will be well maintained. The technicians lack the necessary expertise” (key informant from a sub-city health department, Addis Ababa).*

Refrigerator supplies provide guarantees against technical failures; however, because of poor accountability mechanisms, they do not receive timely maintenance service. In the Gambella and SW regions, the theft of solar panels was pointed out as a challenge. As reported from Afar and Amhara, some of the recently distributed refrigerators are not functioning owing to installation problems or a skill gap among health workers.

#### Frequent power outages and lack of backup power options.

According to EPI focal persons, limited access to reliable electricity remains a challenge for the cold chain system at health facilities. At central and regional EPSS hubs, this is not a concern because dependable backup power is mostly available. Although WoHO, hospitals, and health centers typically have backup generators, the cold chain is sometimes compromised because of the shortage of fuel in the market and lack of budget for fuel purchase. Kerosene-powered refrigerators are not optimally functioning for the same reasons.

“In our hospital, there are [an] adequate number of refrigerators, but we commonly face prolonged power outage[s]. It is not possible to use the generator for 24 hours” (EPI focal person from a primary hospital in the Afar region).

“You can’t get kerosene in the market even if you have the budget” (Maternal and Child Health Directorate Director at a ZHD in the SW region).

### Vaccine wastage.

According to key informants, both open and closed vial wastages are major concerns in Ethiopia. So far, various measures have been taken at different scales to reduce wastage. This includes health workers’ training on the EVM, introduction of a temperature monitoring and reporting system, prioritization of the distribution and use of vaccines based on VVM status, and proactive transfer of near-to-expiry vaccines from one hub to another with the aim of preventing mass wastage. However, the interventions have been met with limited success because of the reluctance and high turnover of health workers, weak vaccine cold chain management at health facilities, and lack of vaccine wastage monitoring and accountability systems.

“I have to be frank here. Vaccine wastage is very high. For example, 50% wastage is expected for BCG, but actually it’s beyond that. It may be up to 60 or 70%” (key informant Sheka ZHD, SW region).

Key informants, including those from the EPSS, recently experienced bulk vaccine wastage due to excess supply, distribution of nearly expired products, interruption of immunization service, and cold chain failure owing to a power outage or failure of refrigerators. During campaigns, the vaccines are distributed in mass to health facilities without optimal forecasting, leading to wastage.

“We don’t have a system to monitor vaccine wastage. We have not assessed vaccine wastage independently for each antigen” (key informant from the Oromia RHB).

“During campaigns, wastage of vaccines is high. I think the main reason for this is ill forecasting” (key informant from the EPSS Assosa hub).

The cold chain system is not closely monitored, as expected, by untrained or ignorant health workers, especially over the weekends. According to a national direction aimed at reducing open vial wastage, health workers should only reconstitute vaccines such as measles and BCG after making sure the minimum required number of children is available. However, this is sometimes violated because of the low turnout for vaccination, fear of missed opportunities, and pressure from caregivers to receive the service. Informants from the community indicated that reluctance of health workers to provide vaccines with the goal of reducing wastage has limited their satisfaction with the service. The multidose PCV, which can be kept refrigerated up to 4 weeks, is also unreasonably wasted because of failure to properly implement the national open vial policy.

“The primary cause of vaccine wastage is negligence of health workers. The refrigerator needs to be checked twice a day. They open [multidose] vials without assessing how many children are available for vaccination” (key informant from the Gambella RHB).

## DISCUSSION

It is clear from the study that the last mile vaccine delivery initiative has improved the Ethiopian VSCLS and enabled the health system to focus on its core functions. All relevant key stakeholders were also positive about the transition. The few available studies in sub-Saharan African countries also indicate that reforms that shortened the supply chain have successfully improved end-to-end vaccine delivery. A simulation study based on Niger’s supply system indicated that bypassing a regional-level intermediary doubled the availability of vaccines at health facilities.^16^ In Legos, Nigeria, evading government areas in the chain has helped to overcome supply side constraints.^17^ In Zambia, a direct distribution system, compared with the traditional three-level system, reduced the frequency and duration of stockouts.^15^ Analysis of the vaccine logistic systems of 57 GAVI-eligible countries suggested the same.^14^

However, last mile delivery success stories appear modest in remote areas where health facilities are still supplied through WoHOs. In many areas, the chain is frequently disrupted by conflicts and flooding, suggesting that the system lacks agility to adapt to disasters. The EPSS may have to consider innovative delivery strategies, such as the use of remotely controlled unmanned aerial vehicles, in such scenarios. Pilot studies have suggested that drones may provide an economical and timely alternative for the delivery of medical supplies, including vaccines, to otherwise inaccessible areas.^25^^,^^26^

Health extension workers need to regularly collect their supplies from nearby health centers, as last mile delivery has not reached the health posts yet. This has caused frequent closure of health posts and demotivation of HEWs. In fact, demotivation of HEWs is a major threat to the Ethiopian flagship Health Extension Program.^27^ Obviously, it’s not practically feasible to the EPSS to directly supply nearly 20,000 health posts in Ethiopia. However, a system that capacitates health centers and allows them to supply their satellite health posts may need to be designed. Such initiative also has the potential to improve linkage within the PHCUs.

In Ethiopia, mass distribution of SDD refrigerators has enabled health posts to provide vaccination service. However, a shortage of functional refrigerators remains a blockade owing to the scarcity of spare parts and skilled technicians. A national SARA concluded that only one-third of health facilities and 14% of health posts in Ethiopia have refrigerators.^16^ A study in the Amhara region of Ethiopia showed that many health facilities have one or more broken refrigerators and that the maintenance system was rudimentary.^28^ Similarly, a survey in seven zones of Ethiopia reported that 32% of health centers and 71% of health posts had nonfunctional refrigerators.^29^ A review of the use of SDD refrigerators in LMICs also identified lack of preventive maintenance, scarcity of qualified technicians, and budget constraints in purchasing spare parts as major bottlenecks.^30^

Globally, digitization is supporting effective distribution of vaccines. Studies from LMICs indicate that technologies, including mobile-based applications, have simplified the logistics system and reduced supply hiccups. In India, an electronic vaccine intelligence network has abridged stockouts and vaccine wastage.^31^ In Tanzania, a digital logistics information platform with an imbedded stock notification system increased the availability of vaccines at service delivery points.^32^ In Nigeria, a digital vaccine stock management tool considerably reduced stockout alerts^33^ According to our findings, a locally developed mobile stock management tool has simplified the vaccine information system and increased vaccine availability. However, the system has not been sustained. This demonstrates the need for understanding the hurdles facing the Ethiopian VSCLS with the use of digital innovations.

The study suggested that vaccine wastage is a concern in Ethiopia and is caused by multifaceted factors, including the reluctance of health workers, ill-forecasting during campaigns, cold chain problems, and lack of vaccine wastage monitoring and accountability systems. In particular, open vial wastage of lyophilized vaccines (measles and BCG) is reportedly common. In fact, vaccine wastage is a global problem that has not received adequate attention thus far.^34^ A study that evaluated aspects of vaccine wastage in Ghana, Mozambique, and Pakistan concluded that wastage was common and was caused by a lack of knowledge and poor tracking systems.^35^ For lyophilized vaccines, decreasing doses per vial could reduce waste, but the low storage capacity may limit use of the smaller dose formats.^36^

Respondents from the community identified bottlenecks that had relevance for EPI service provision, including 1) service interruption due to disruptions in vaccine supply; 2) limited availability of static service at health posts due to a shortage of refrigerators; and 3) hesitancy of health workers to provide lyophilized vaccines fearing wastage. Unless promptly addressed, the gaps may lead to demand-side barriers and compromise national coverage and equity goals. With the goal of balancing vaccine wastage and service satisfaction, the MoH may need to introduce a lower-dose format for BCG and measles.

The peculiar strength of the study is that it captured the perspectives of all relevant stakeholders of the national VSCLS and explored critical points along the entire supply chain. However, the following limitation needs to be considered when interpreting the findings. Because the study focused primarily on remote and underserved settings, over-identification of the supply hiccups cannot be ruled out. Community-level interviewees were selected by engaging local HEWs, and this may theoretically alter the findings of the study. Some of the reported VSCLS bottlenecks (e.g., frequency of supply interruptions, extent of vaccine wastage) could have been assessed more effectively with a mixed-methods design. Finally, the study did not assess the cost-effectiveness of the last mile delivery approach, and economic evaluation of the initiative needs to be considered if it has to be replicated in other countries.

## CONCLUSION

The last mile delivery system has improved the national VSCLS and enabled the health sector to focus on strategic activities. However, in remote areas the system’s reach remains modest, threatening national coverage and equity goals. The VSCLS has not established alternatives for ground transportation for reaching remote and conflict-affected areas. Other blockades include the unavailability of refrigerators at health posts, lack of an effective refrigerator maintenance system, weak supply linkage within the PHCU, lack of backup power at health centers, scarcity of refrigerated tracks, and unmanageably large catchment areas for EPSS hubs. Vaccine wastage appears to be common and is caused by multifaceted problems. So far, the Ethiopian VSCLS has not sustainably embraced digital logistics solutions.

The readiness of the health posts to provide regular vaccination services has to be ensured by making refrigerators universally available. To ensure uninterrupted availability of vaccines at health posts, district health offices and health centers need to be capacitated. Efforts should be made to adopt, scale up, and sustain digital technologies for improving the vaccine logistics system. The effectiveness of unmanned aerial vehicles to reach remote and conflict-affected areas needs to be piloted. Vaccine wastage should be reduced by improving the cold chain system and instating monitoring and accountability mechanisms. Future decisions on adopting new vaccines should take the VSCLS into consideration. Without significant investments to modernize the national VSCLS, the aspiration to make EPI universally accessible will remain unrealistic.

## Supplemental Materials

10.4269/ajtmh.23-0622 Supplemental Materials
