# Supplementary material for: Perspectives on the Performance of the Ethiopian Vaccine Supply Chain and Logistics System after the Last Mile Delivery Initiative: A Phenomenological Study
Source: Am J Trop Med Hyg. 2024 Apr 2;110(5):1029–38. doi: 10.4269/ajtmh.23-0622 (PMC11066354; doi:10.4269/ajtmh.23-0622)
Supplement: Supplemental Materials [file tpmd230622.SD1.pdf]

Supplementary Table 1: List of KIIs and FGDs

| Federal-level respondents (# 14 KII) |
|---|
| - MoH, MCH Directorate Deputy Director<br>- MoH, EPI Team Leader<br>- MoH, Primary Health Care (PHC) Technical Advisor<br>- MoH, Policy and Planning, Monitoring and Evaluation Directorate Director<br>- MoH, Health System and Special System Team Leader<br>- MoH, Disease Surveillance and Response Directorate Director<br>- EPHI, Disease Surveillance and Response Directorate<br>- Federal EPSA, Vaccine Supply Manager<br>- EPI Focal persons at core partner organizations (UNICEF, WHO, CHAI, PATH) |
| Regional-level respondents (# 84 KII) |
| - RHBs, MCH Director<br>- RHBs, EPI Team Leader or equivalent<br>- RHBs, HEP Director<br>- Regional EPSA Hubs, EPI Focal Person<br>- Regional EPHI, PHEM Director<br>- Local partners (2/region): UNICEF, WHO, CDC, JSI, CHAI, Save the Children, Transform PHCU |
| Zonal or sub-city level respondents (# 36 KII) |
| - MCHN Directorate Director<br>- EPI Team leader |
| Woreda-level respondents (# 66 KII) |
| - MCHN Directorate Director<br>- EPI Team Leader<br>- HEP Directorate Director<br>- Local implementing partners/ NGOs |
| Primary Health Care Unit (PHCU) (# 88 KII) |
| - MCH/EPI head (Primary hospital)<br>- HEW supervisors (health centers)<br>- MCH/EPI head (health centers)<br>- HEWs |
| Community-level respondents (# 39 KII, 22 FGDs) |
| - Kebele administrators<br>- Influential community members<br>- Women Development Army/Health Development Army (WDA/HDA)<br>- Local women/caregivers |
